# *Rotundedessa* gen. n., a New Edessinae Genus with the Description of Two New Species from Argentina (Hemiptera: Pentatomidae)

**DOI:** 10.1007/s13744-026-01417-3

**Published:** 2026-08-04

**Authors:** María Cecilia Martínez, Freddy Burgos Gallardo, José Antonio Marin Fernandes, Luiz Alexandre Campos

**Affiliations:** 1 Consejo Nacional de Investigaciones Científicas y Técnicas (CONICET), Instituto de Biología de la Altura, Univ Nacional de Jujuy, S. S. de Jujuy, Jujuy, Argentina; 2 Cátedra de Ordenamiento Ambiental, Facultad de Ciencias Agrarias, Univ Nacional de Jujuy, Jujuy, Argentina; 3 Instituto de Ciências Biológicas, Univ Federal do Pará, Belém, Pará Brazil; 4 Depto de Zoologia, Instituto de Biociências, Univ Federal do Rio Grande do Sul, Porto Alegre, Rio Grande do Sul Brazil

**Keywords:** New taxa, Neotropical region, *Edessa*, Argentina

## Abstract

The Edessinae is a Neotropical subfamily of Pentatomidae comprising hundreds of species in 20 genera. The nominal genus *Edessa* Fabricius, 1803 includes most of this diversity with approximately 350 nominal species, and can be considered a “depot taxon.” Studying small groups of morphologically similar species is an efficient approach to deal with the taxonomic diversity in *Edessa* and has led to the description of several new genera. The *viridula* group comprises five species and is characterized by short metasternal processes, wrinkled ventrolateral areas, and pygophore with several unique features. Following the current standards in the taxonomy of Edessinae, we consider these characteristics distinctive enough to propose a new genus to the species included in the *viridula* group. The new genus is herein described and new combinations for the five species formerly placed in the *viridula* group are proposed. Additionally, two new species from Argentina are described, and a key to the species of the new genus is given.

## Introduction

The Edessinae is a Neotropical group of pentatomids (Hemiptera: Pentatomidae) characterized by astonishing diversity, being the second largest subfamily in number of species, and comprising almost 500 species currently classified into 20 genera. The nominal genus *Edessa* Fabricius, 1803 includes approximately 350 nominal species, with around 300 yet to be described (Mendonça et al. 2023b). This genus can be considered a “depot of species,” and has its limits historically confounded with those of Edessinae (Grazia et al. 2015). To tackle the taxonomic organization of such immense diversity straightforwardly, an efficient approach that has been used is to study small groups of morphologically similar species (e.g., *stalii* group, Campos et al. 2020; *ovina* group, Fernandes and Silva 2021; *sexdens* group, Mendonça et al. 2023a, b). This approach has led to the elevation of several former subgenera to genus (e.g., *Pygoda* Amyot & Serville, Fernandes et al. 2018; *Hypoxys* Amyot & Serville, Nunes et al. 2020) and the description of new genera (e.g., *Calcatedessa* Silva and Fernandes 2021; *Odara* Campos and Fernandes 2023).

The *viridula* species group was proposed for five South American species that share short metasternal processes, wrinkled ventrolateral areas, and a short pygophore, leaving the parameres and the proctiger placed close to the pygophore walls (Nascimento et al. 2017). Considering that the subgenus *Edessa* was recently characterized (Mendonça et al. 2023a), and that the species considered here do not fit into any current subgenera (both under review, Fernandes, pers. comm.), we hereby propose a new genus to accommodate the species of the *viridula* group. We also describe two new species from Argentina, and update the identification key provided by Nascimento et al. (2017).

## Materials and Methods

Labels of the name-bearing types are quoted verbatim (following, e.g., Kment et al. 2018): a slash (/) is used to divide data on different lines of one label, a double slash (//) to divide data on different labels, and authors’ comments are given in square brackets ([]). Measurements in millimeters (minimum – maximum) were taken for the following morphometric parameters: total length (from the apex of mandibular plates to the apical angles of urosternite VII), length and width of the head (including eyes), pronotum and scutellum, length of the antennomeres, and body maximum width at urosternite III. Photographs of the specimens and their genital structures were taken using a Nikon AZ100M stereomicroscope equipped with a DS-Fi2 digital camera. The images were processed using the NIS Elements AR Microscope Imaging software to create a single focused photograph, which in turn was edited using GIMP (version 3.0.4). Coordinates (latitude, longitude) of the collection sites of the specimens are in decimal degrees.

The male pygophore was relaxed using hot water, and then removed using entomological pins and forceps. The terminology follows Zhou and Rédei (2020) for female genital structures, and Kment and Vilímová (2010) for the external scent efferent system of the metathoracic glands. The type specimens were deposited in the Museo de La Plata (MLP) and the Instituto de Biología de la Altura (INBIAL), Argentina.

## Results and Discussion

***Rotundedessa*** **Martínez,** **Fernandes & Campos, gen. n.**

*viridula* group, Nascimento et al. 2017: 137.

**Etymology**. The name refers to edessines of a round-shaped body. Latin: rotundus, round. Noun in apposition, feminine.

**Type species**. *Edessa viridula* Nascimento et al. 2017.

**Description**. The original description of the *viridula* group presented in Nascimento et al. (2017) is sufficient to characterize and identify the genus, as follows. Small species (10.4–13.7 mm), with oval and depressed body; dorsal surface distinctly flat; uniformly light green, including corial veins and genital segments, except median ventral abdominal yellow area; punctures dense and concolorous. Antennae green or light brown. Humeral angles rounded and not developed. Evaporatorium with distinct transversal ridges; lateral area contiguous to metapleural evaporatoria wrinkled and punctured. Legs light brown. Lateral third of abdominal sternites wrinkled. Pygophore with vestigial superior process of the genital cup, reduced to a line or row of dark brown or black tiny tubercles; parameres robust and short; and ventral rim of pygophore with a small translucent flap at the bottom of the median excavation.

The following characteristics are added to the genus description. Abdominal trichobothria placed on yellow calli. Pygophore with a densely setose and short genital cup, leaving the proctiger on the edge of the ventral rim. Dorsal rim fused to the posterolateral angles. Parameres with all lobes rounded and swollen, the dorsal lobe larger than the others, the two ventral lobes not on the same level, globular, with one usually projecting posteriorly. Ventral rim with a dense tuft of setae on each side of the proctiger, connected by a conspicuous keel (reduced only in *R. eucnema*).

Included species: *Rotundedessa angusticlada* (Nascimento et al. 2017) comb. n.; *Rotundedessa eucnema* (Nascimento et al. 2017) comb. n.; *Rotundedessa exigusternata* (Nascimento et al. 2017) comb. n.; *Rotundedessa rugulata* (Nascimento et al. 2017) comb. n.; *Rotundedessa viridula* (Nascimento et al. 2017) comb. n.; *Rotundedessa luteola* Martínez, Fernandes & Campos, sp. n.; and *Rotundedessa olivacea* Martínez, Fernandes & Campos, sp. n.

**Comments**. See Nascimento et al. 2017.

**Distribution**. Brazil, Bolivia, Argentina (NEW RECORD).

***Rotundedessa luteola*** **Martínez,** **Fernandes & Campos, sp. n.**

**Etymology**. The name refers to the yellowish apex of scutellum. Latin: luteolus, yellowish.

**Holotype**. Male. ARGENTINA, Jujuy/Puesto Viejo – Holcim [600 m.a.s.l.]/−24.467769 −64.8999110 (WGS 84)/18-III-2025/Burgos Gallardo F. col. (9610. INBIAL).

**Paratype**. Female. Same data as the holotype (he – 18627. MLP).

**Measurements**. Head length (1.6–1.7), width (2.2–2.4); pronotal length (2.6–2.9), width (6.4–6.5); scutellar length (4.7–5.0), width (4.3–4.5); length of antennal segments: I (0.5–0.6), II (0.8–1.0), III (1.0–1.3), IV (1.3–1.4), V (1.6); total length (7.4–9.8); abdominal width (6.7–7.0).

**Fig. 1:**
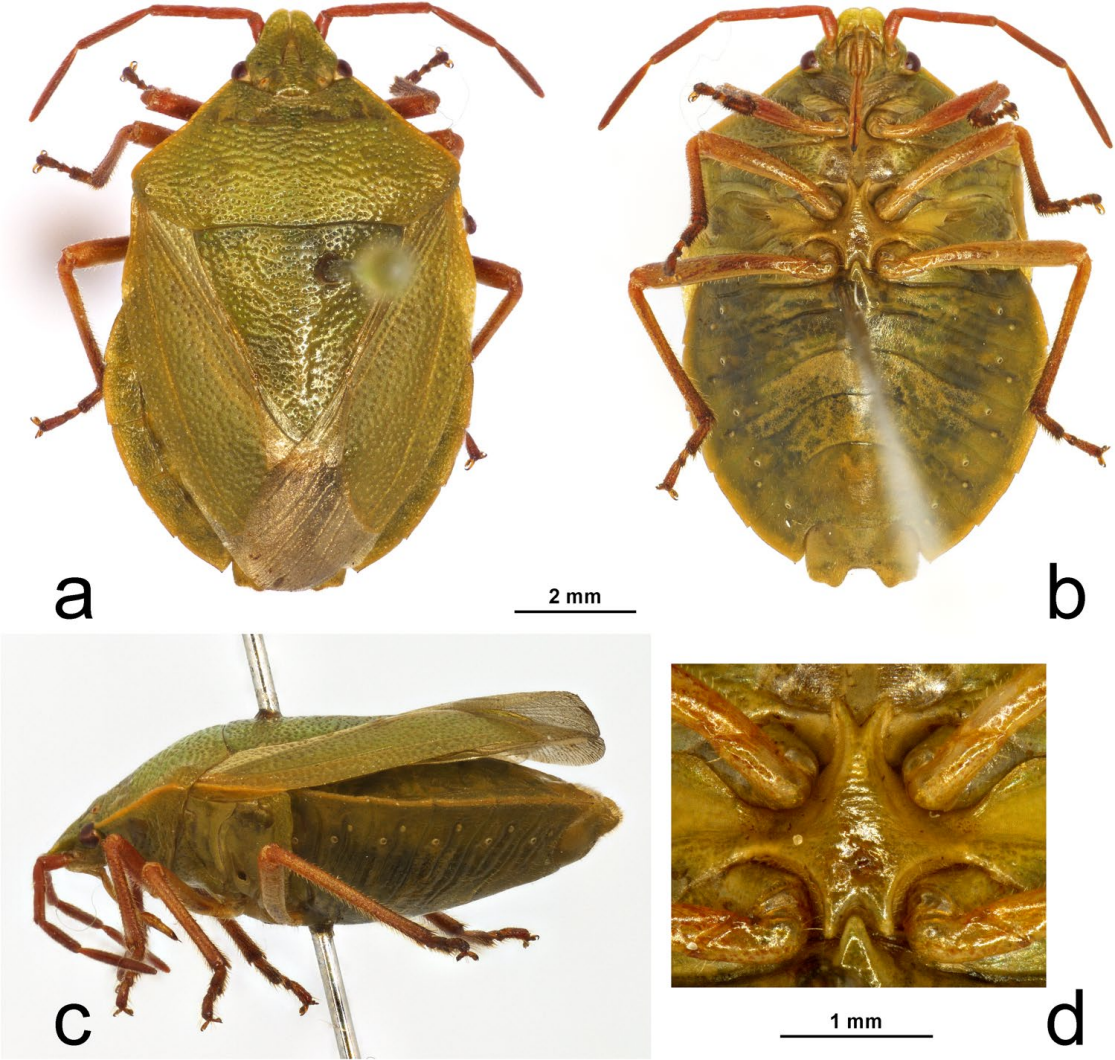
*Rotundedessa luteola* Martínez, Fernandes & Campos, sp. n., holotype (male). **a**–**c** Dorsal, ventral, and lateral, respectively, all in the same scale. **d** Metasternum, ventral

**Description**. Body light green, bordered in yellow, except the head that is uniformly green. Apex of scutellum with yellowish callus. Pronotum and scutellum shallowly rugose. Rostrum not attaining the metasternal process. Arms of the anterior bifurcation of the metasternal process short, little surpassing the suture of the mesocoxal socket (Fig. 1). Peritreme not reaching half the distance between the scent gland ostiole and lateral margin of the body (Fig. 5a). Contiguous margin of meso and metapleura raised. Spiracles yellowish. Antennae, rostrum, and legs reddish brown, spotless.

**Fig. 2:**
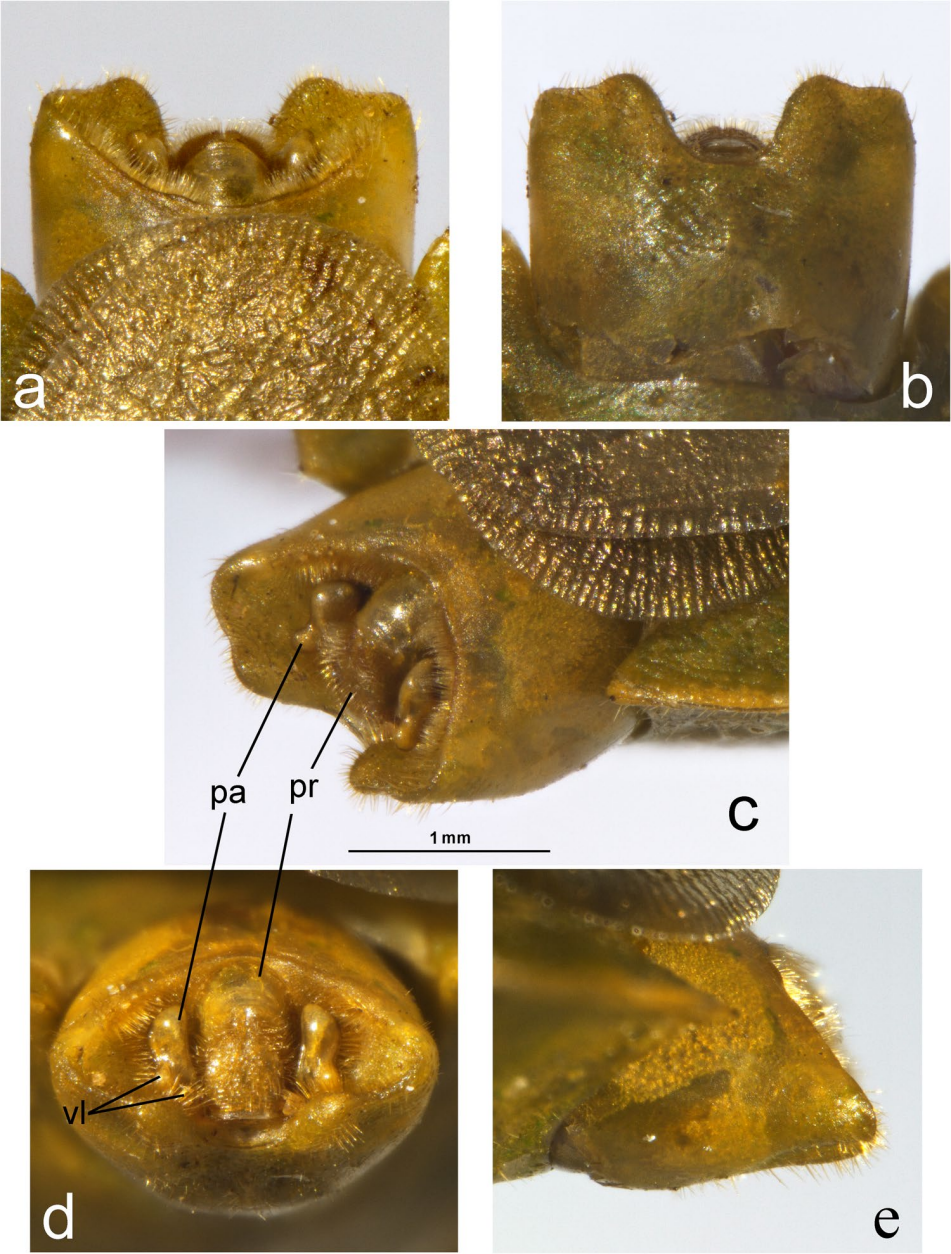
*Rotundedessa luteola* Martínez, Fernandes & Campos, sp. n., holotype (male), pygophore, **a–e** dorsal, ventral, dorsolateral, posterior, and lateral, respectively. pa, paramere; pr, proteger; vl, ventral lobes of paramere

**Male**. Ventral part of the paramere with a short round and a long digitiform lobes, base wider than distal part; digitiform lobe distinctly projected posteriorly (Fig. 2).

**Fig. 3:**
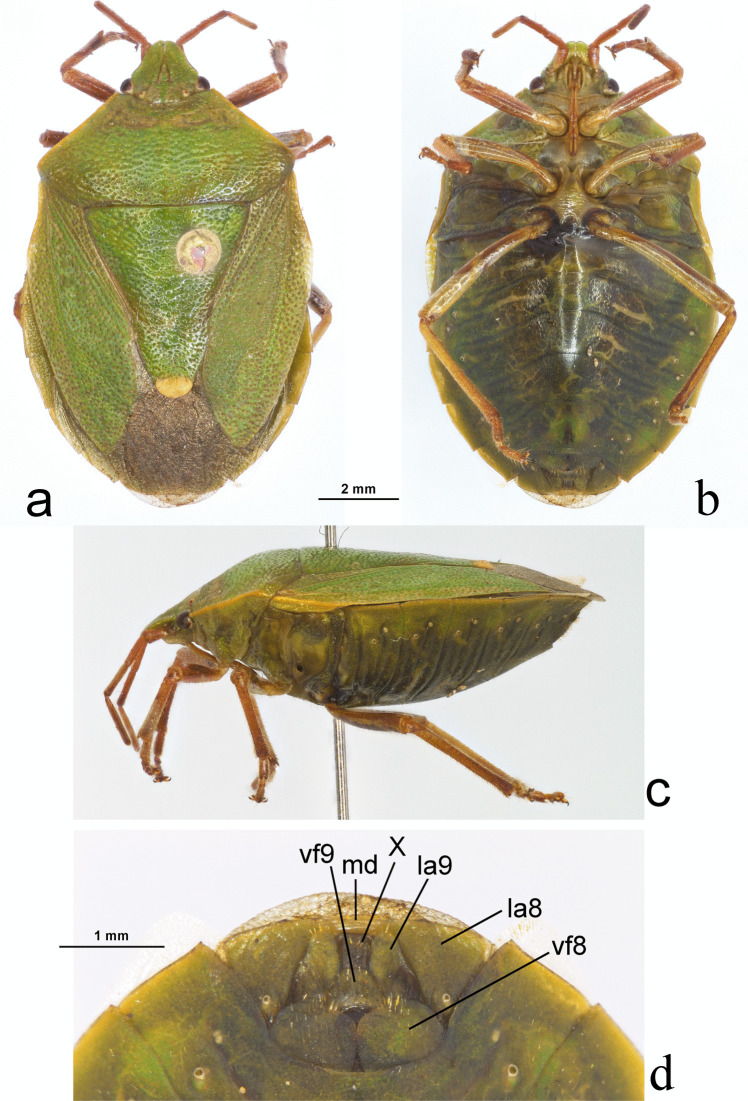
*Rotundedessa luteola* Martínez, Fernandes & Campos, sp. n., paratype (female). **a**–**c** Dorsal, ventral, and lateral, respectively, all in the same scale. **d** Genital plates, ventro-posterior. la8, laterotergite 8; la9, laterotergite 9; md, mediotergite 8; vf8, valvifer 8; vf9, valvifer 9; X, segment X

**Female**. Genital plates setulose. Posterior margin of valvifers 8 convex, more projected posteriorly over laterotergites 9; sutural margins partially juxtaposed, slightly divergent proximally and distally. Laterotergites 9 with base excavated, apex round, not attaining the posterior margin of mediotergite 8 (Fig. 3).

**Comments**. This species shares with *R. viridula* the peritreme length, almost reaching half the distance between the ostiole and lateral margin of the body. The shape of the paramere is also similar in both species. On the other hand, *Rotundedessa luteola* is clearly distinguishable from the others by the unique yellow spot on the scutellum apex, rostrum not reaching the anterior bifurcation of the metasternal process, and metasternal process short with the anterior arms poorly developed, just surpassing the mesosternal limit.

**Distribution**. Argentina (Jujuy) (Fig. 7).

***Rotundedessa olivacea*** **Martínez,** **Fernandes & Campos, sp. n.**

**Etymology**. The name refers to the olive-green body color. Latin: oliva, olive.

**Holotype**. Male. ARGENTINA, Salta/G. [General] Güemes - Quisto-/Finca San Luis [1807 m] – Filo Pastizal/10-I-2024/Martínez M.C. col. [−24.6200, −64.6536] (9609. INBIAL).

**Paratype**. Male. ARGENTINA, Jujuy/[San Juan de Dios] Estancia Las Lauras [974 m.a.s.l.]/−24.564629 −64.651530 (WGS 84)/27-XI-2024/Martínez M. C. col. (7817. INBIAL).

**Measurements**. Head length (1.4–1.6), width (2.2–2.4); pronotal length (2.6–2.7), width (6.4–6.6); scutellar length (4.8–5.1), width (4.0–4.1); length of antennal segments: I (0.7–0.9), II (1.08–1.1), III (1.2–1.3), IV (1.5–1.9), V (1.9–2.3); total length (11.1–11.3); abdominal width (6.4–6.7).

**Fig. 4:**
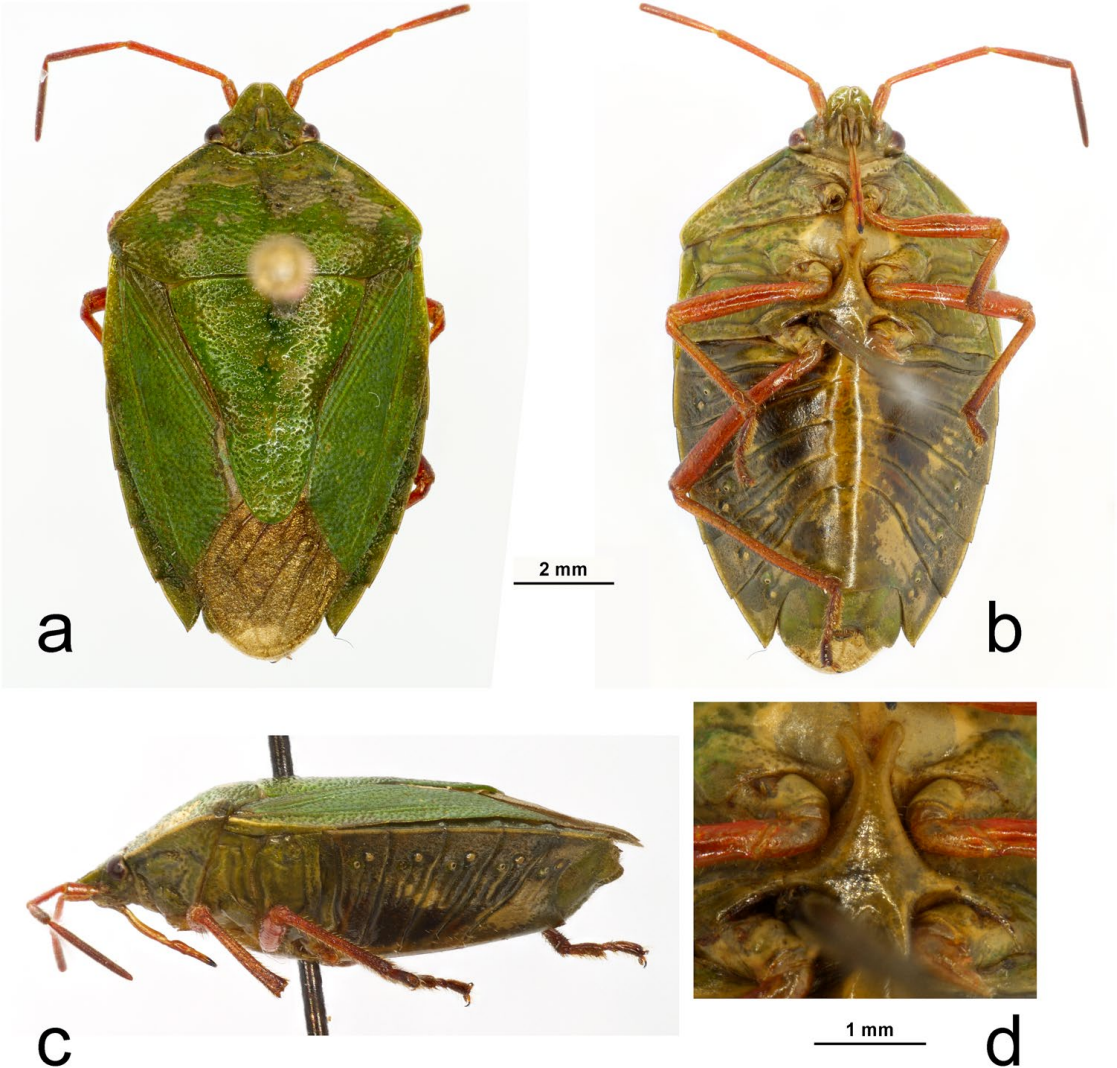
*Rotundedessa olivacea* Martínez, Fernandes & Campos, sp. n., holotype (male). **a**–**c** Dorsal, ventral, and lateral, respectively, all in the same scale. **d** Metasternum, ventral

**Fig. 5:**
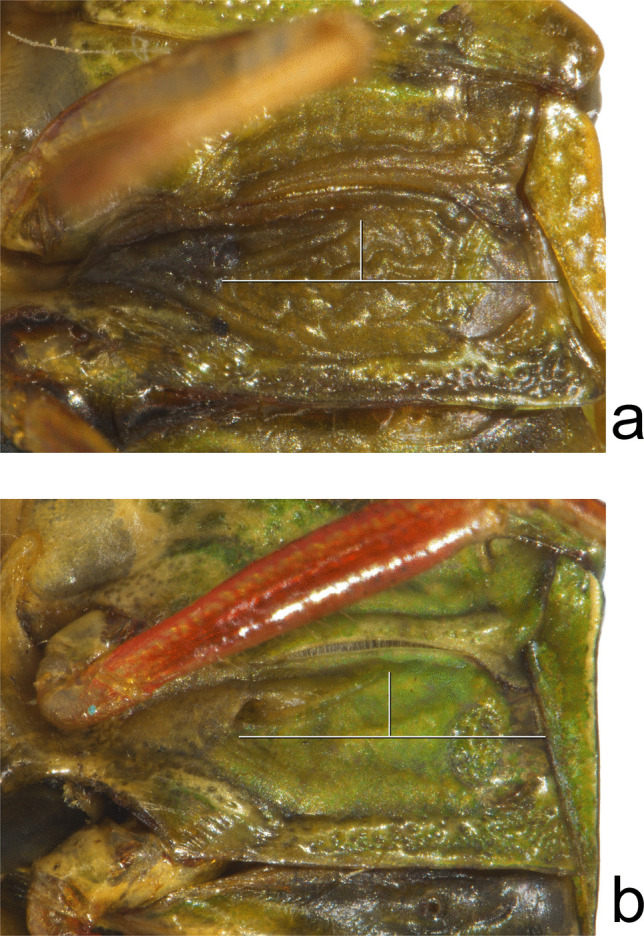
External scent efferent system. **a** *Rotundedessa luteola* Martínez, Fernandes & Campos, sp. n., paratype (female). **b** *Rotundedessa olivacea* Martínez, Fernandes & Campos, sp. n., holotype (male)

**Description**. Body olive green; pronotal anterolateral margins, mandibular plates, and ventral lateral margin of urosternites bordered in green. Pronotum and scutellum shallowly rugose, almost smooth. Rostrum attaining the metasternal process. Arms of the anterior bifurcation of the metasternal process almost reaching half the length of the swollen portions of mesosternum (Fig. 4). Peritreme reaching half the distance between the scent gland ostiole and lateral margin of the body. Evaporatorium with a low crest in mesopleura delimited by sulci (Fig. 5b); contiguous margin of meso and metapleura also raised; metapleura with two raised crests, one medial and the other distal. Spiracles yellowish; trichobothria placed in light yellow callus. Antennae, rostrum, and legs reddish brown, spotless.

**Fig. 6:**
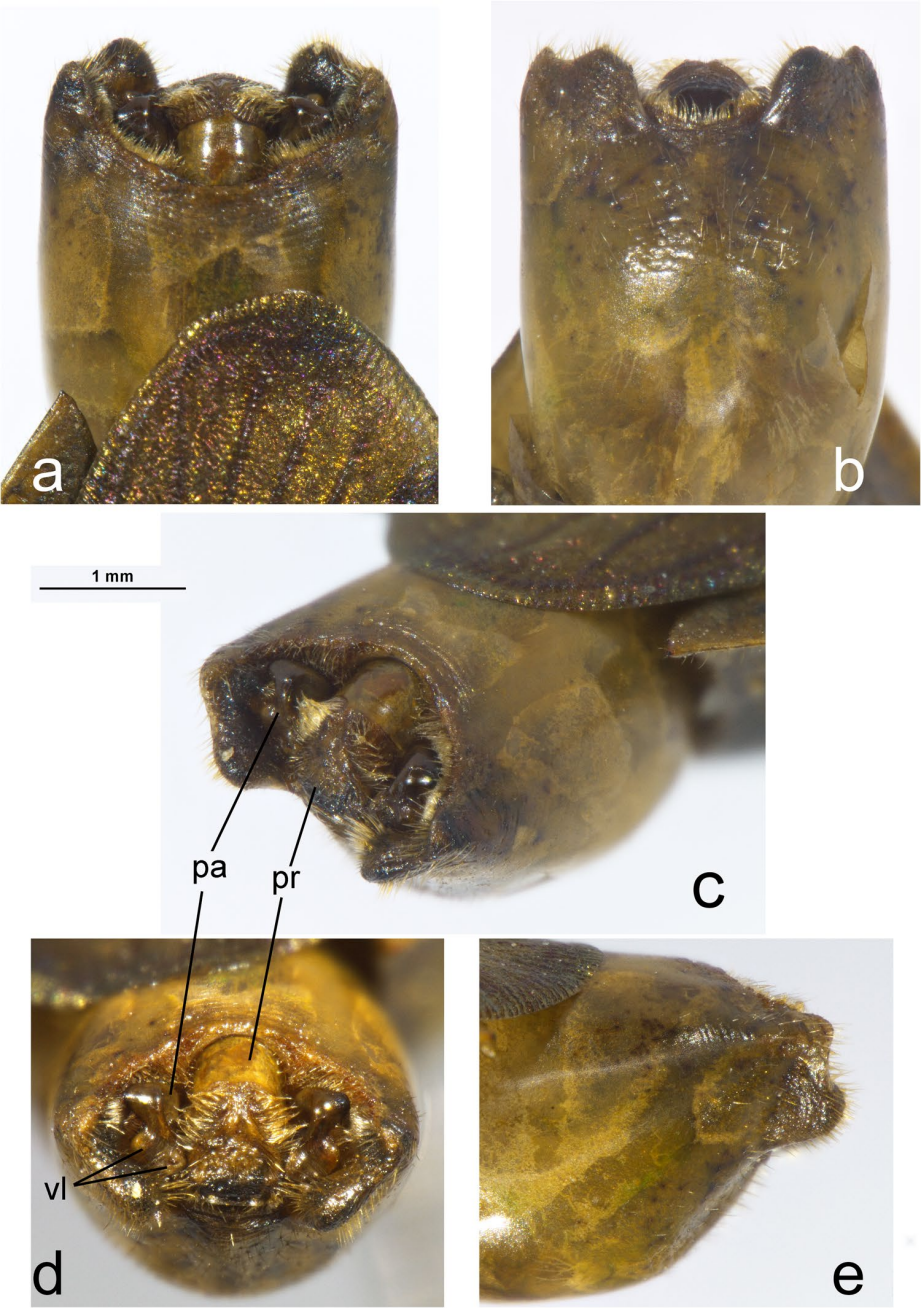
*Rotundedessa olivacea* Martínez, Fernandes & Campos, sp. n., holotype (male), pygophore, **a–e** dorsal, ventral, dorsolateral, posterior, and lateral, respectively. pa, paramere; pr, proctiger; vl, ventral lobes of paramere

**Male**. Ventral part of the paramere with two short round lobes, the lateral lobe tumescent and slightly longer than the median lobe; base narrower than distal part (Fig. 6).

**Female**. Unknown.

**Comments**. This species is similar to *R. eucnema*, both share the paramere broader distally than basally. Nonetheless, *Rotundedessa olivacea* has the posterolateral angles of the pygophore more developed; folded parameres; and outer ventral lobe of the paramere less developed than in *R. eucnema*.

**Fig. 7:**
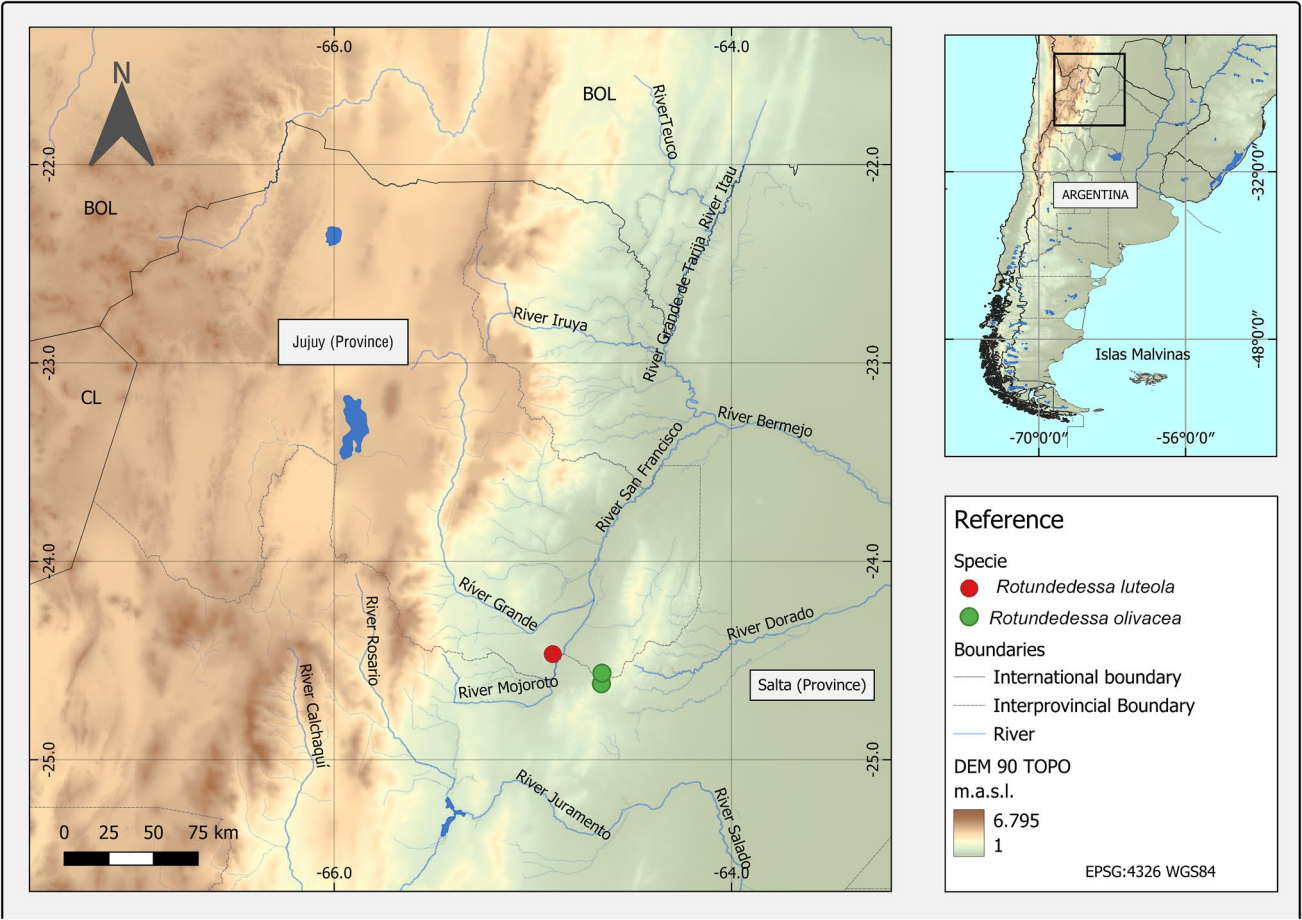
Collection sites of *Rotundedessa luteola* Martínez, Fernandes & Campos, sp. n., and *Rotundedessa olivacea* Martínez, Fernandes & Campos, sp. n.

**Distribution**. Argentina (Jujuy, Salta) (Fig. 7).

### Identification key to the species of ***Rotundedessa*** (adapted from Nascimento et al. 2017)

1 Metasternal process with anterior bifurcation small and barely developed; spiracles black … *R. exigusternata *

- Metasternal process with anterior bifurcation clearly developed; spiracles variable, never contrastingly black … 2

2 Spiracles yellowish, contrastingly lighter than surrounding area; costal angle of coria rests on connexivum VII … 3

- Spiracles concolorous with surrounding area, if lighter colored the costal angle of coria rests on connexivum VI … 4

3 Metasternal arms short, only reaching the swollen portion of mesosternum; rostrum not attaining metasternum … *R. luteola *Martínez, Fernandes & Campos, sp. n.

- Metasternal arms almost reaching half the length of the swollen portion of mesosternum; apex of rostrum resting between the arms of metasternum … *R. olivacea *Martínez, Fernandes & Campos, sp. n.

4 Antenna and legs with tiny and sparse brown dots; metasternal process with laterally inclined arms receiving only apex of the last rostral segment … *R. eucnema *

- Antenna and legs spotless; metasternal process with not laterally inclined arms receiving half or more of the fourth rostral segment … 5

5 Peritreme short, almost reaching 1/3 of the distance between ostiole of the scent gland and lateral margin of the body; arms of the anterior bifurcation of metasternal process shorter than fourth rostral segment and tumid distally … *R. rugulata*

- Peritreme long, about half of the distance or more between ostiole of the scent gland and lateral margin of the body; arms of the anterior bifurcation of metasternal process not tumid distally and as long as fourth rostral segment … 6

6 Peritreme almost reaching half of the distance between ostiole of the scent gland and lateral margin of the body; posterolateral angles of the pygophore developed; paramere with both ventral lobes developed; gonocoxites 8 with outline clearly arched; laterotergites 9 narrow … *R. viridula*

- Peritreme clearly surpassing half of the distance between ostiole of the scent gland and lateral margin of the body posterolateral angles of the pygophore not developed; paramere with both ventral lobes not developed; gonocoxites 8 with outline slightly arched; laterotergites 9 broad … *R. angusticlada*
